# A Comprehensive Interaction Network Constructed Using miRNAs and mRNAs Provides New Insights into Potato Tuberization under High Temperatures

**DOI:** 10.3390/plants13070998

**Published:** 2024-03-30

**Authors:** Ming He, Ju Liu, Jie Tan, Yinqiao Jian, Jiangang Liu, Yanfeng Duan, Guangcun Li, Liping Jin, Jianfei Xu

**Affiliations:** 1 Key Laboratory of Biology and Genetic Improvement of Tuber and Root Crops, Ministry of Agriculture and Rural Affairs, Beijing 100081, China; heming@caas.cn (M.H.); liuju413@163.com (J.L.); tanjie_caas@163.com (J.T.); jianyinqiao@caas.cn (Y.J.); liujiangang@caas.cn (J.L.); duanyanfeng@caas.cn (Y.D.); liguangcun@caas.cn (G.L.); jinliping@caas.cn (L.J.); 2 State Key Laboratory of Vegetable Biobreeding, Institute of Vegetables and Flowers, Chinese Academy of Agricultural Sciences, Beijing 100081, China

**Keywords:** potato, tuberization, high temperature, RNA sequencing, miRNA sequencing

## Abstract

High temperatures delay tuberization and decrease potato (*Solanum tuberosum* L.) yields. However, the molecular mechanisms and regulatory networks underlying tuberization under high temperatures remain largely unknown. Here, we performed the mRNA and miRNA sequencing of leaves and stems to identify genes and regulatory networks involved in tuberization under high temperatures. A total of 2804 and 5001 differentially expressed genes (DEGs) under high-temperature stress were identified in leaves and stems, respectively. These genes were significantly enriched in gene ontology terms regarding meristem development, the sucrose biosynthetic process, and response to heat. Meanwhile, 101 and 75 differentially expressed miRNAs (DEmiRNAs) were identified in leaves and stems, respectively. We constructed an interaction network between DEmiRNAs and DEGs, identifying 118 and 150 DEmiRNA–DEG pairs in leaves and stems, respectively. We found three miRNA–mRNA candidate modules involved in tuberization under high temperatures, including *stu-miR8030-5p*/*StCPY714*, *stu-miR7981f-p5*/*StAGL8a*, and *stu-miR10532A*/*StAGL8b*. Our study constructed an interaction network between miRNAs and target genes and proposes candidate miRNA–gene modules that regulate tuber formation under high temperatures. Our study provides new insights for revealing the regulatory mechanism of the high-temperature inhibition of tuberization and also provides gene resources for improving the heat tolerance in potatoes.

## 1. Introduction

Potato (*Solanum tuberosum* L.) is a critical tuber crop worldwide and is rich in nutrients such as starch, vitamins, and protein. Temperature is one of the most important environmental factors affecting tuber development in potatoes. The 14–22 °C range suits tuber initiation [1]. High temperatures delay or even impede tuber development, particularly tuber formation, causing a decline in tuber yield [2,3,4,5].

Potato tuberization is regulated by a series of tuberigen formation factors. StSP6A is the pivotal tuberization regulator homologous to *Arabidopsis* FLOWERING LOCUS T (FT) [6,7]. StSP6A proteins were synthesized in leaves and transported to stolon to induce tuber formation [6]. The expression of *StSP6A* is inhibited by *SELF-PRUNING 5G* (*StSP5G*), another homolog of *FT*, which is positively regulated by CONSTANS-LIKE 1 (StCOL1) [8]. Silence of either *StSP5G* or *StCOL1* induces tuber formation on non-inductive long days [9]. StSP6A and StFDL1 interact with St14-3-3 directly, comprising the tuberigen activation complex (TAC) [10]. StCEN and StSP6A are competitively combined with StFDL1 to suppress tuberization [11]. StBRC1b inhibits StSP6A activity and represses tuberization in aerial axillary bubs by interacting directly with StSP6A, which in turn limits sucrose accumulation and StSP6A transporting to axillary buds, thereby promoting tuberization underground [12]. In addition to *StSP6A*, *StSP3D*, *StFTL1*, *microRNA156* (*miR156*), and *miR172* function as mobile signals in promoting tuberization [13,14,15]. All tuberization regulatory pathways converge to *StSP6A* and participate in regulating tuber formation by regulating the expression level of *StSP6A*.

A recent study proposed that post-transcriptional regulation plays a significant role in the early stages of tuberization [5]. In *miR156*-overexpressed potato plants, which present increased aerial tubers, *StBRC1b* expression was inhibited. In contrast, the loss of *StBRC1b* function leads to an upregulated *miR156* expression, indicating that the inhibition of aerial tuberization might be regulated through the negative regulation of the *StBRC1b* expression by *miR156* [12]. At elevated temperatures, the expressions of *StSP5G* and *StCOL1* are upregulated, suppressing the expression of *StSP6A* [5,16,17]. miRNA suppressing the expression of SP6A (*SES*), which targets *StSP6A*, was induced during high-temperature stress. Interfering with the function of *SES* promotes tuberization under high temperatures [4]. *miR172* is a stimulating tuberization factor [18,19]. *miR156*-resistant *SQUAMOSA PROMOTER BINDING-LIKE* (*StSPL9*) overexpression lines increase *miR172* abundance, suggesting a *miR156*/*StSPL9*/*miR172* regulatory module in tuberization [19]. Therefore, post-transcriptional regulation also plays an important role during tuber formation in addition to transcriptional regulation.

miRNAs play essential roles in regulating the protein accumulation of target transcripts during high-temperature stress in plants. A large number of high-temperature-related miRNAs have been reported, such as *miR156*, *miR172*, and *miR824* [20,21,22,23]. In alfalfa, the overexpression of *miR156* improves heat tolerance, paralleling the downregulation of its target gene, *Squamosa Promoter-Binding Protein-Like* 13 (*SPL13*) [21,24]. The abundance of *miR172* is elevated during heat stress, inducing the expression of *FT* and flowering in *Arabidopsis* [20,25]. The MADS-box transcription factor *AGAMOUS LIKE 16* (*AGL16*), a flowering negative regulator, is negatively regulated by *miR824*, leading to a mild depression of *FT* [22,26]. However, the function of miRNAs in tuberization during high-temperature stress remains unclear.

In this study, high-temperature stress was induced in vitro to investigate the high-temperature response mechanism during tuberization in potatoes. We performed mRNA and miRNA sequencing and identified differentially expressed genes and miRNAs in leaves and stems between normal and high temperatures. An enrichment analysis identified regulatory pathways during tuberization under high temperatures. A comprehensive interaction network was constructed under high temperatures between miRNAs and target genes for tuberization. We identified key candidate miRNA–target gene pairs involved in tuberization under high-temperature stress. Our study shows a comprehensive understanding of tuberization under high temperatures in potatoes.

## 2. Materials and Methods

### 2.1. Plant Materials and Growth Conditions

We used diploid RH89-039-16 (RH, *Solanum tuberosum* L.) in this study, which was preserved at the Institute of Vegetable and Flower Research, Chinese Academy of Agricultural Sciences. The plants were propagated in vitro using single-node stems on MS medium supplemented with 30 g/L sucrose and 7 g/L plant agar under 16 h light/8 h dark at 20 °C. For the tuberization assay in vitro, the single-node stems of RH plants were cultured in MS medium supplemented with 60 g/L sucrose and 7 g/L plant agar under 16 h light/8 h dark at 20 °C for three weeks. Then, the plants were transferred to 8 h light/16 h dark at 20 °C and 27 °C, respectively. The light intensities in all conditions were 2000 lx. Each experiment was designed with three biological replicates of at least 24 plantlets (3 plantlets in each culture bottle). Seedlings with similar plant heights and stem diameters were selected for further culture to avoid the influence of individual plant differences.

### 2.2. mRNA Library Construction and Sequencing

Potato tuberization was delayed about 8 days at 27 °C compared to that at 20 °C in our study. Therefore, leaves and stems of 7-week-old plants with tubers cultured at 20 °C and without tubers cultured at 20 °C and 27 °C were selected for RNA sequencing. The leaves and stems of plants with tubers at 27 °C were not used in this study. The samples of leaves of plants with tubers at 20 °C, leaves of plant without tubers at 20 °C, stems of plant with tubers at 20 °C, stems of plant without tubers at 20 °C, leaves of plant without tubers at 27 °C, and stems of plant without tubers at 27 °C were abbreviated as TL_20, UTL_20, TS_20, UTS_20, UTL_27, and UTS_27, respectively. Each sample was designed with three biological replicates. One sample of UTL_20 was abolished due to its poor uniformity with the other two samples in the principal component analysis.

Total RNA was extracted using TRIzol reagent (Invitrogen, Carlsbad, CA, USA) following the manufacturer’s procedure. The RNA of each sample was quantified using the Agilent Bioanalyzer 2100 system. Poly(A) RNA was purified using Dynabeads Oligo(dT) (Thermo Fisher, Waltham, CA, USA) and then fragmented into small pieces using a Magnesium RNA Fragmentation Module (NEB, Boston, USA). The RNA fragments were reverse transcribed to create cDNA using SuperScript™ II Reverse Transcriptase (Invitrogen, cat. 1896649, USA). Then, the U-labeled second-stranded DNAs were synthesized with DNA polymerase I (NEB, cat.m0209, USA), RNase H (NEB, cat.m0297, USA), and dUTP Solution (Thermo Fisher, cat. R0133, USA). The blunt ends of each strand were added an A-base. The fragments were ligated with single- or dual-index adapters and underwent size selection with AMPureXP beads. Finally, we performed sequencing on an Illumina Novaseq™ 6000 (LC-BIO Technology CO., Ltd., Hangzhou, China) following the manufacturer’s protocol.

### 2.3. Mapping and Analysis of RNA Sequencing Data

The RH genome from http://solanaceae.plantbiology.msu.edu/RH_potato_download.shtml (accessed on 28 January 2021) was used as the reference genome. The Cut adapt software (https://cutadapt.readthedocs.io/en/stable/, cutadapt-1.9) was used to obtain clean data to remove the low-quality bases and undetermined bases with adaptors, the proportion of N (N represents uncertain base information) greater than 5%, or Q ≤ 10 accounts for more than 20% of the entire read. Then, the clean data were mapped to the RH genome using HISAT2 (https://daehwankimlab.github.io/hisat2/, hisat2-2.0.4) to obtain a bam file with parameters of ~hisat2-1 R1.fastq.gz-2 R2.fastq.gz-S sample_mapped.sam. The StringTie software (http://ccb.jhu.edu/software/stringtie/, stringtie-1.3.4d. Linux_x86_64) was used for the assembly of genes or transcripts with parameters of ~stringtie -p 4 -G genome.gtf -o output.gtf -l sample input.bam. The final annotation was obtained using gffcompare software (http://ccb.jhu.edu/software/stringtie/gffcompare.shtml, gffcompare-0.9.8. Linux_x86_64). The FPKM was quantified according to the balltown package with parameters of ~stringtie -e -B -p 4 -G merged.gtf -o samples.gtf samples.bam. Differentially expressed genes (DEGs) were identified using DESeq2 (http://www.bioconductor.org/packages/release/bioc/html/DESeq2.html) [27]. Parameters of |log2(Fold Change)| ≥ 1, *p*-value < 0.05, and q-value < 0.05 were used to retrieve the DEGs.

### 2.4. miRNA Library Construction, Sequencing, and Analysis

The samples for miRNA analysis were isolated from the total RNA used for RNA sequencing. MiRNAs were isolated from the total RNA via agarose gel electrophoresis. The library construction was conducted according to the standard steps provided by Illumina Company. TruSeq Small RNA Sample Prep Kits (Illumina, San Diego, CA, USA) were used to prepare the libraries. The constructed libraries were sequenced using Illumina Hiseq2500 at LC-BIO (Hangzhou, China). MiRNA sequences were identified using ACGT101-miR (LC Sciences, Houston, TX, USA). Unique sequences with 18–25 nucleotides in length were mapped to potato precursors in miRBase 22.0 using BLAST search to identify known miRNAs and novel 3p- and 5p-derived miRNAs. In the alignment, length variation at both 3′ and 5′ ends and one mismatch within the sequence were allowed. Unique sequences that matched mature miRNAs from specific species in the hairpin arm were identified as known miRNAs. Unique sequences that matched the other arm of the known precursor hairpin arm of a specific species (opposite to the annotated arm containing mature miRNAs) were considered novel 3p- and 5p-derived miRNAs. The naming of miRNAs refers to the miRBase database (What’s in a name? http://www.mirbase.org/blog). For the miRNAs that did not have an annotation before in potato, we named them according to the homologous miRNAs in other species. The expressions of miRNAs were calculated as reported by Li et al. 2016 [28]. The differentially expressed miRNAs (DEmiRNAs) were screened out with parameters of a *p*-value < 0.05 and q-value < 0.05.

### 2.5. Principal Component, Gene Enrichment, and Hierarchical Clustering Analysis

A principal component analysis was performed using the vegan package of R. A gene ontology enrichment analysis, KEGG pathway enrichment analysis, and hierarchical clustering analysis of the DEGs and DEmiRNAs were performed using TBtools (version 1.0.99) [29].

### 2.6. miRNA–mRNA Interaction Network Generation

To identify the potential miRNA–mRNA network for tuberization during high temperatures, we used the DEmiRNAs and their target DEGs for the interaction network. The putative target genes of miRNAs were predicted using psRobot (http://omicslab.genetics.ac.cn/psRobot/, v1.2) with parameters of score ≤ 2.5. The graphic network was created using Cytoscape 3.9.1 [30].

### 2.7. Quantitative Real-Time PCR Analysis

The mRNA and miRNA validation were performed according to the manufacturer’s protocol (Hieff^®^ qPCR SYBR^®^ Green Master Mix, YEASEN; miRNA Universal SYBR qPCR Master Mix, Vazyme, Nanjing, China), and the template-specific primers were designed using Primer3.0 (https://primer3.ut.ee/) and miRNA Design V 1.01 software (Vazyme). All reactions were conducted in a Light Cycler 480 Real-Time PCR System (Roche Life Science, Basel, Switzerland). Reaction specificity was assessed using a melting curve, and qPCR data were analyzed using the 2^−ΔΔCt^ method. The relative expression level was calculated relative to *EF1α* and *U6* snRNA. All primer sequences for the real-time PCR analysis are listed in Table S2.

## 3. Results

### 3.1. Suppression of Potato Tuberization In Vitro under High Temperatures

**Figure 1:**
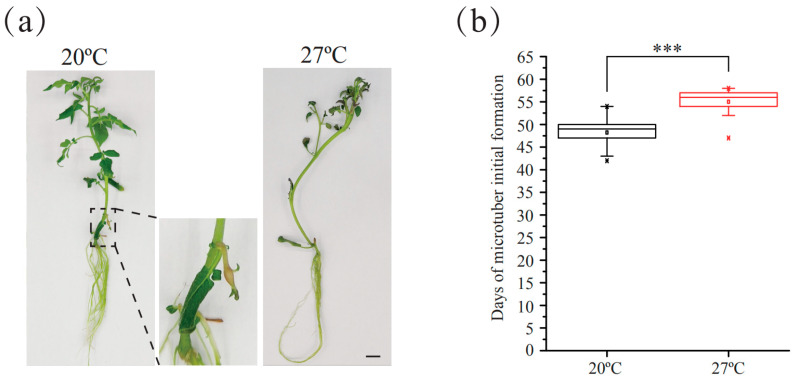
High temperature suppresses tuberization in vitro. (**a**) Forty-nine-day-old potato plants in vitro. Scar bar = 1 cm. (**b**) Boxplots show microtuber initial formation time at different temperatures. *** *p* < 0.001, Student’s *t* test.

To explore the mechanism of potato tuberization under high temperatures, we induced high-temperature stress in vitro for a phenotypic analysis. Plants were cultured at a normal temperature of 20 °C for three weeks under long days (LDs) (16 h light/8 h dark). Then, the plants were transported to 27 °C and 20 °C under short days (SDs) (8 h light/16 h dark) for subsequent growth. We observed and determined the tuberization time to evaluate the effect of high-temperature stress. The average initiation tuberization time under 27 °C was 55 days, which was significantly delayed compared to 20 °C (47 days; Figure 1).

### 3.2. Transcriptome Analysis of Tuberization under High-Temperature Stress

RNA sequencing was performed to investigate the molecular basis of tuberization under high-temperature stress in potatoes. Considering that the expressions of many regulators for tuberization were controlled in leaves, but the proteins were transported to the stolon through stems to regulate tuberization, leaves and stems were both sequenced. At least two biological replicates of each tissue were used to construct the cDNA libraries. A total of 87.28 Gb valid data were generated after conducting a quality control of the raw sequencing reads, with an average mapped ratio of 96.29%. The Q30 was 98.42%, and the GC content was 43.03% (Table S1). We randomly selected 10 genes for real-time PCR (RT-PCR) to verify the accuracy of the transcriptome data (Table S2). The R^2^ between RNA-seq and RT-qPCR was estimated to be 0.8184, which illustrates the reliability of our transcriptome (Figure S1).

**Figure 2:**
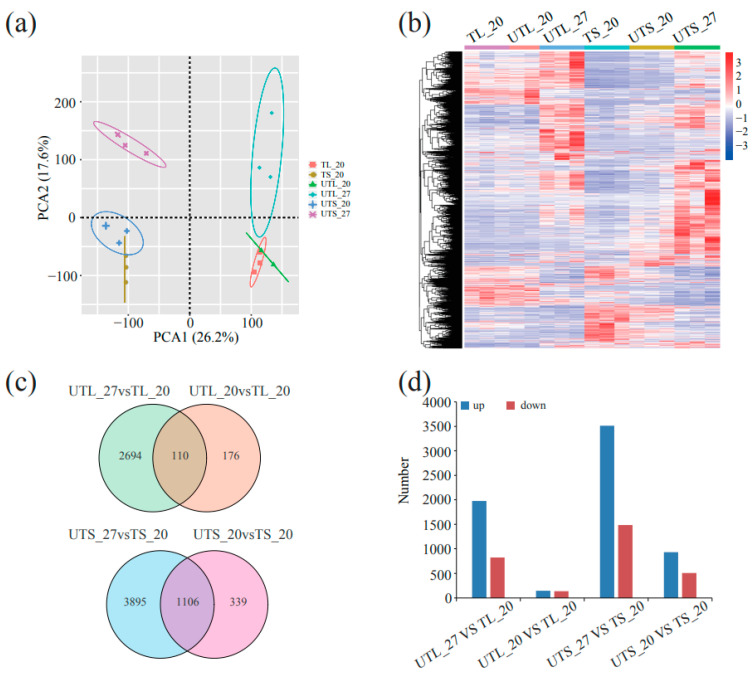
Transcriptome analysis of leaves and stems under different temperatures. (**a**) Principal component analysis showing the relationships of samples in leaves and stems at 20 °C and 27 °C. The X-axis and Y-axis represent PCA1 and PCA2, respectively, explaining 46.1 and 43.8% of the total variance. (**b**) Hierarchical cluster analysis of DEGs. The columns and rows represent samples and DEGs, respectively. The color key on the right shows normalized FPKM values for relative gene expressions. (**c**) Venn diagram of DEGs at different temperatures. The upper and lower rows represent samples of leaves and stems, respectively. (**d**) Quantification of upregulated and downregulated genes at high temperature of 27 °C compared to normal temperature of 20 °C. TL_20, UTL_20, TS_20, UTS_20, UTL_27, and UTS_27 represent leaves of plant with tubers at 20 °C, leaves of plant without tubers at 20 °C, stems of plant with tubers at 20 °C, stems of plant without tubers at 20 °C, leaves at 27 °C, and stems at 27 °C, respectively.

The principal component analysis of the gene expression dataset showed high uniformity between biological replicates (Figure 2a). The clusters of leaves and stems at 20 °C (TL_20, leaves of plants with tubers at 20 °C; UTL_20, leaves of plant without tubers at 20 °C, TS_20, stems of plant with tubers at 20 °C; UTS_20, stems of plant without tubers at 20 °C) were distinguished clearly from those at 27 °C (UTL_27, leaves at 27 °C; UTS_27, stems at 27 °C). These results were further supported through the hierarchical clustering analysis, which showed similar gene expression profiles at the same temperatures but significantly distinct gene expression profiles between different temperatures (Figure 2b).

The differentially expressed genes (DEGs) were analyzed by comparing UTL_27 vs. TL_20, UTL_20 vs. TL_20, UTS_27 vs. TS_20, and UTS_20 vs. TS_20. A total of 2804 and 286 DEGs were identified compared to UTL_27 vs. TL_20 and UTL_20 vs. TL_20, respectively (Figure 2c). Among 2804 DEGs, there were 1977 up- and 827 downregulated genes (Figure 2d). Only 110 DEGs were related to both comparisons, UTL_27 vs. TL_20 and UTL_20 vs. TL_20, most of which showed similar expression patterns between UTL_27 and UTL_20 (Figure S2).

For the stem, 5001 and 1445 DEGs were identified by comparing UTS_27 vs. TS_20 and UTS_20 vs. TS_20, respectively (Figure 2c). In total, 1106 genes overlapped between these two comparisons (Figure 2c) and showed similar expression patterns between UTS_27 and UTS_20 (Figure S3a). These genes were significantly enriched regarding biological process gene ontology (GO) terms related to cell wall biogenesis-related processes (Figure S3b).

### 3.3. Gene Ontology Annotation and Pathway Enrichment Analysis of DEGs

**Figure 3:**
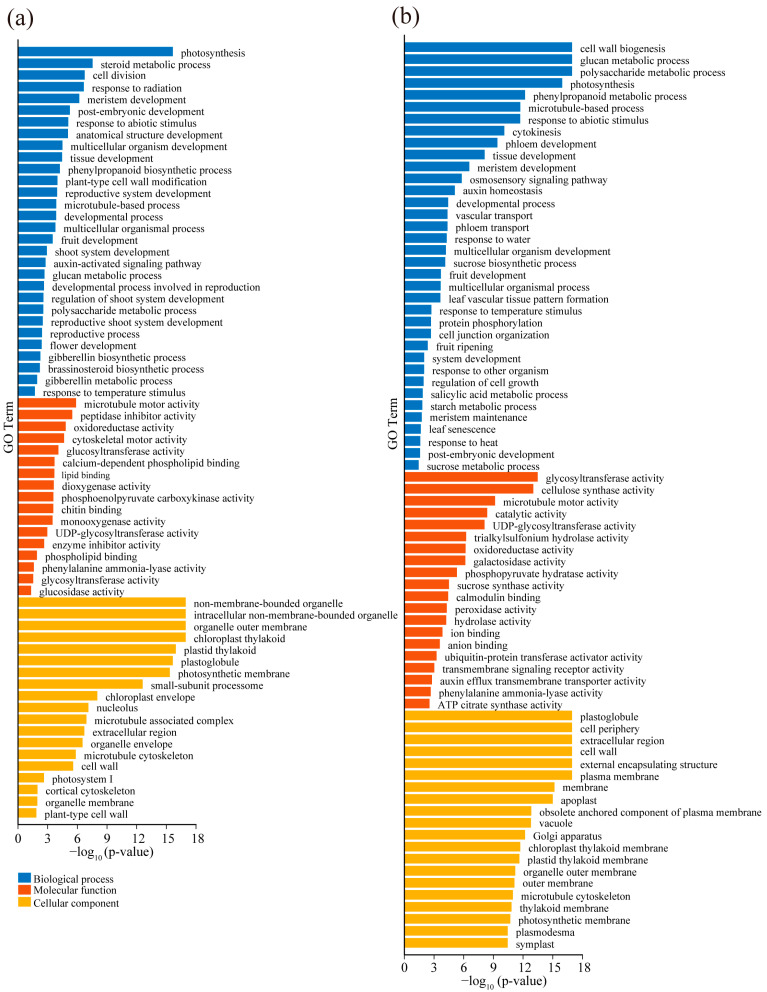
Gene ontology (GO) enrichment analysis of DEGs under high-temperature stress. GO term enrichment analysis of DEGs in leaves (**a**) and stems (**b**). The colors of the box represent −log_10_ (*p*-value). The size of the boxes represents the gene count for GO terms. Biological process, molecular function, and cellular component terms are presented in blue, red, and yellow, respectively.

We performed GO term enrichment analysis on the total DEGs in comparison of UTL_27 vs. TL_20 and UTS_27 vs. TS_20 to comprehensively classify the functional categories of the DEGs. The DEGs, in comparison of UTL_27 vs. TL_20, were significantly enriched in biological process (BP) terms related to photosynthesis, cell division, meristem development, tissue development, and gibberellin metabolic process (Figure 3a). In the molecular function (MF) category, GO terms, such as microtubule moto activity, glucosyltransferase activity, and enzyme inhibitor activity, were significantly enriched (Figure 3a). In the cellular component (CC) category, the DEGs were enriched regarding the chloroplast thylakoid, chloroplast thylakoid, and cell wall (Figure 3a). The DEGs in comparison of UTS_27 vs. TS_20 were enriched in the developmental process, sucrose biosynthetic process, and response to heat (Figure 3b). In the MF category, GO terms, such as glycosyltransferase activity, calmodulin binding, and the activity of ubiquitin-protein transferase activator, were significantly enriched (Figure 3b). In the CC category, the DEGs were enriched regarding the plastoglobule, cell periphery, and cell wall related terms (Figure 3b).

We performed a pathway enrichment analysis based on the KEGG database to further analyze the molecular response under high-temperature stress. Leaf DEGs were significantly enriched in amino acid, fructose, and mannose metabolism related terms (Figure S4a). Stem DEGs were significantly enriched in 27 pathways, for instance, membrane trafficking, MAPK signaling pathway, amino acid metabolism, environmental information processing, and signal transduction (Figure S4b).

### 3.4. Analysis of Typical Tuberization Regulators under High Temperatures in Potatoes

**Figure 4:**
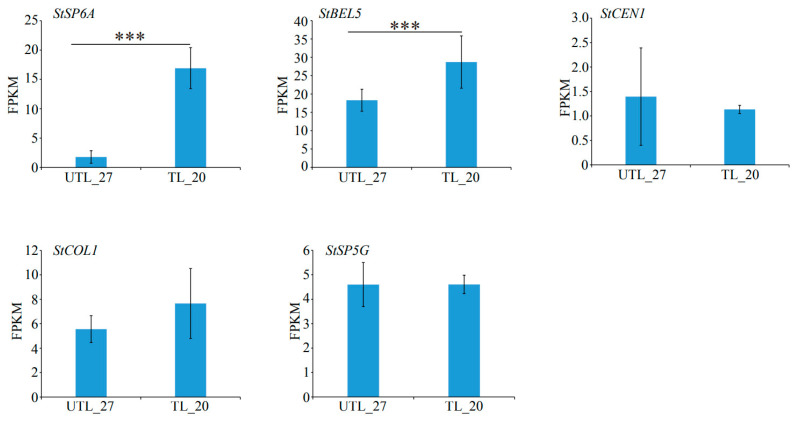
Expression of tuberization-related genes. The FPKM statistics of genes related to tuberization in leaves at different temperatures. Error bars indicate ± SD. *** *p* < 0.001, Student’s *t* test.

Tuberization is regulated by a series of tuberigen formation factors, such as *StSP6A*, *StBEL5*, *StSP5G*, *StCEN1*, and *StCOL1*, and forms a regulatory pathway centered around *StSP6A* [31]. We found that the expressions of *StSP6A* (RHC05H1G2713) and *StBEL5* (RHC06H2G2699) were significantly downregulated at 27 °C compared to 20 °C. The expressions of *StSP5G* (RHC05H2G2263), *StCEN1* (RHC03H1G1254), and *StCOL1* (RHC02H2G2633) were not obviously altered at 27 °C (Figure 4), which is similar to that reported by Park et al. (2022) [5]. These results suggest that the regulatory mechanisms of tuberization at high temperatures might differ from those at normal temperature.

### 3.5. Expression Dynamics of miRNAs during High-Temperature Stress

**Figure 5:**
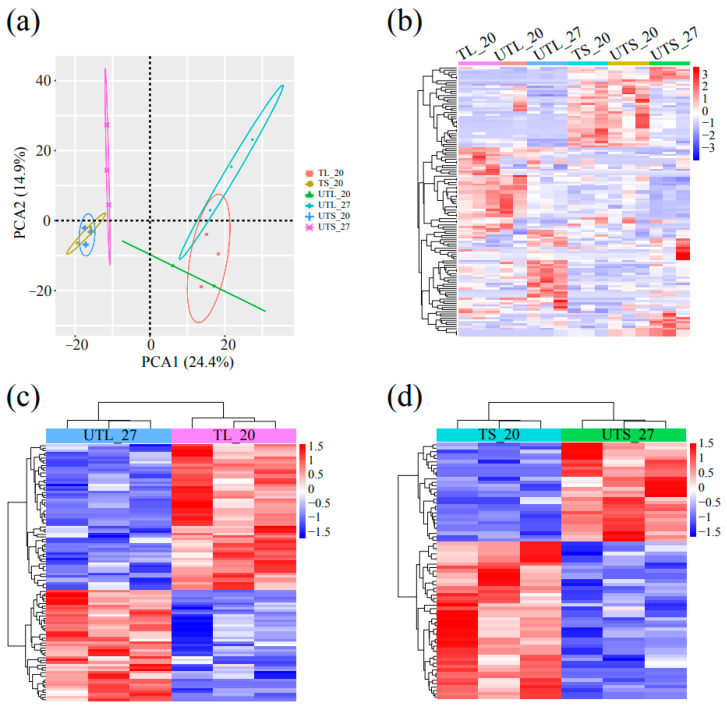
miRNA sequencing analysis of leaves and stems under different temperatures. (**a**) Principal component analysis showing the relationships of samples in leaves and stems under different temperatures. The X-axis and Y-axis represent PCA1 and PCA2, respectively, explaining 24.4 and 14.9% of the total variance. (**b**) Hierarchical cluster analysis of total miRNAs. (**c**,**d**) Hierarchical cluster analysis of DEmiRNAs in leaves and stems, respectively. The columns and rows represent samples and miRNAs, respectively. The color key on the right of (**b**–**d**) shows normalized norm values for relative expression.

Post-transcriptional regulation plays an essential role at the early stage of tuberization, such as microRNA (miRNAs, miR) regulation [5]. We performed miRNA sequencing to identify the miRNAs involved in tuber formation during high temperatures. The miRNA expression was validated using RT-PCR and was consistent with miRNA sequencing, suggesting the reliability of miRNA sequencing (Figure S5 and Table S2). The principal component analysis showed that clusters of leaves and stems at 20 °C were distinguished clearly from those at 27 °C, similar to the RNA sequencing analysis (Figure 5a). These results were supported by the hierarchical clustering analysis (Figure 5b).

A total of 463 miRNAs belonging to 75 families were identified (Figure S6 and Table S3), among which 200 miRNAs were known in potato (Table S4). In comparison of UTL_27 vs. TL_20, 104 differentially expressed miRNAs (DEmiRNAs) were identified, including 45 up- and 59 downregulated miRNAs (Figure 5c). A total of 1913 target genes were predicted, most of which were expressed differently between TL_20 and UTL_27 (Figure S7a). These 1913 target genes were enriched in BP terms regarding the defense response, response to stress, and cell death (Figure S7b). Similarly, 75 DEmiRNAs in comparison of UTS_27 vs. TS_20 were identified, including 29 upregulated and 46 downregulated miRNAs (Figure 5d), and 1275 target genes were predicted and expressed differently between TS_20 and UTS_27 (Figure S7c). All the target genes were significantly enriched in BP terms related to the cellular response to stimulus, the regulation of hydrolase activity, and anther morphogenesis (Figure S7d).

### 3.6. Regulatory Networks between DEGs and DEmiRNAs under High Temperatures

**Figure 6:**
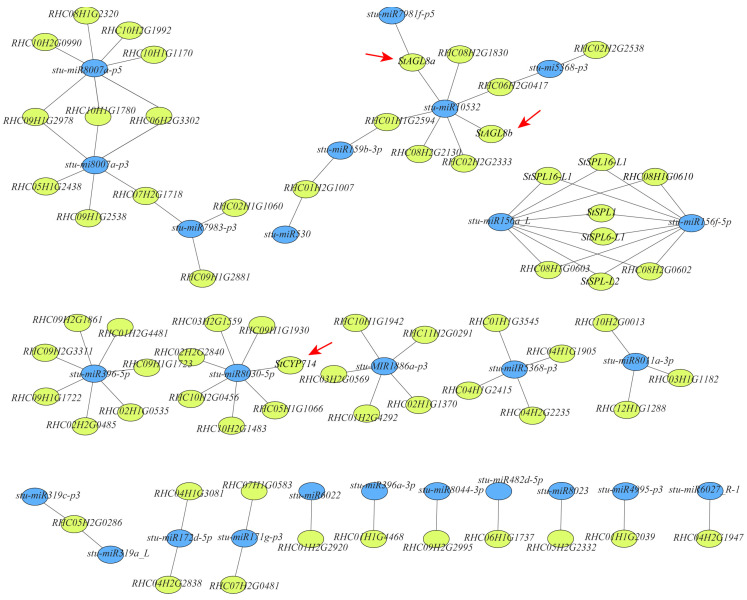
DEmiRNA–DEG interaction networks in leaves. The DEmiRNA–DEG interaction network was built using Cytoscape 3.9.1. Blue circles represent DEmiRNAs; yellow circles represent target genes. The candidate pairs of *stu-miR8030-5p*/*StCPY714*, *stu-miR7981f-p5*/*StAGL8a*, and *stu-miR10532A*/*StAGL8b* are labeled with red arrows.

We constructed interaction networks using DEmiRNAs and DEGs to identify the potential miRNAs and target genes correlated with tuberization under high temperatures. In total, 118 DEmiRNA–DEG pairs were identified in the leaf (Figure 6 and Table S5). Among these pairs, five *StSPLs* were determined to be targeted by *stu-miR156f-5p* and *stu-miR156a_L*, of which their expressions were reduced under high temperatures (Figure 6 and Figure S8). Among these five *StSPLs*, the expressions of *StSPL6*-*like1* (*StSPL6*-*L1*), *StSPL6*-*like2* (*StSPL6-L2*), *StSPL16*-*like1* (*StSPL16-L1*), and *StSPL16*-*like2* (*StSPL16-L2*) in leaves increased under high temperatures (Figure S9). Two MADS translation factors RHC06H2G2343 and RHC06H1G2441, named *StAGL8a* and *StAGL8b*, respectively, were found to be targeted by *stu-miR7981f-p5* and *stu-miR10532A* (Figure 6). The orthologous genes of these factors in Arabidopsis and tomato play crucial roles in floral organ development and fruit ripening [32,33,34]. The expression levels of these two genes at 27 °C were significantly reduced compared with those at 20 °C (Figure S9). In addition, a P450 monooxygenase *RHC03H2G1398.2*, named *StCPY714*, was found to be a target of *stu-miR8030-5p* and downregulated in leaves under high temperatures (Figure 6 and Figure S9). The homologous genes of *StCPY714* in Arabidopsis and rice catalyze the deactivation of bioactive gibberellins, which have been reported to be negative regulators during tuberization [35]. These DEmiRNA–DEG pairs were considered candidate regulators for tuberization under high temperatures. In addition, we found that the *miR172d_5p* expression increased under high temperatures and targeted two endoglucanase genes (RHC04H1G3081 and RHC04H2G2838; Figure 6 and Figure S8).

**Figure 7:**
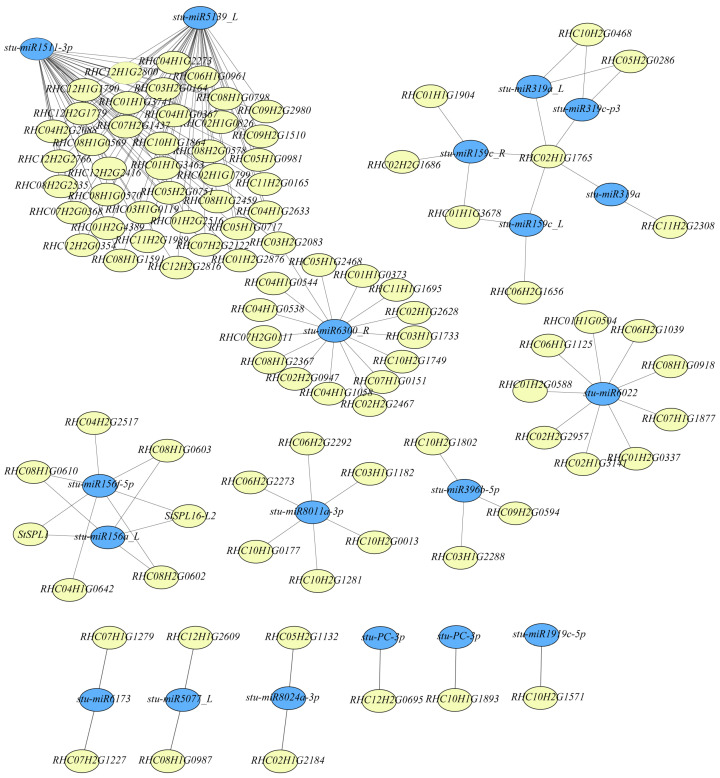
DEmiRNA–DEG interaction networks in stems. The DEmiRNA–DEG interaction network was built using Cytoscape 3.9.1. Blue circles represent DEmiRNAs; yellow circles represent target genes.

For stems, 150 pairs of DEmiRNA–DEGs were identified, among which 13 pairs comprising 5 DEmiRNAs and 9 DEGs overlapped with those in leaves, including *stu-miR156f-5p* and *stu-miR156a_L* (Figure 7 and Table S6). However, only two *StSPLs* (*StSPL1* and *StSPL6-L2*) targeted by *miRNA156* were identified in the stem due to the similar expression levels of other *StSPLs* at 20 °C and 27 °C (Figure S9), indicating different regulation mechanisms between leaves and stems in response to high temperatures.

## 4. Discussion

Potato tuberization is regulated by environmental signals and internal factors, such as temperature and hormones [16,35]. In this study, we demonstrated high-temperature delays in tuberization in vitro. RNA sequencing was performed to investigate the regulatory pathways for tuberization under high temperatures. We identified 2804 DEGs in leaves under high temperatures, most of which were enriched in GO terms related to temperature stress-related processes, development-related processes, and hormone response processes (Figure 3a). Meanwhile, 5001 DEGs were identified in stems, which were significantly enriched in processes related to sucrose metabolism, hormone response, and tissue development (Figure 3b). These results comprehensively reveal the pathways of tuberization under high-temperature stress.

The expression of *StSP6A* is controlled by the negative regulator *StSP5G*, which is positively regulated by *StCOL1* [8]. StSP6A, StFDL1, and St14-3-3 combine to form a tuberigen activation complex to induce tuber formation [10]. StCEN suppresses tuberization by competitively binding StFDL1 through St14-3-3 in TAC [11]. As previously reported [4,5,16,17], our study also found that the expression of *StSP6A* is significantly suppressed under high temperatures. However, the expressions of *StCEN*, *StCOL1*, and *StSP5G* were not altered (Figure 4). Additionally, *StSP6A* transcriptional activity is linked to the expression of *StBEL5*, another tuberization inducer [14]. The *StBEL5* expression was reduced under high temperatures (Figure 4). This result further supports the study by Park et al. (2022) [5] in that other negative regulators exited during tuberization under high temperatures.

miRNAs are essential in plant growth, development, environmental adaptability, and stress resistance. At present, many miRNAs have been reported to respond to temperature signals, such as *miR156*, *miR172*, *miR824*, and *miR168* [20,21,22,23]. In rice and cassava, the expression of *miR156* is reduced under high-temperature stress [36,37]. An elevated expression of *miR156* in alfalfa enhances the response to high temperatures, paralleling the reduced expression of target gene *SPL13* [21,24]. In potatoes, the overexpression of *miR156* suppresses tuberization, accompanied through reductions in *miR172* levels and *StSP6A* expression and through the suppression of the expression of *StSPLs* [18,19,38]. In our study, 104 DEmiRNAs were identified in leaves under high temperatures, including *miR156* and *miR17*. In total, 1913 target genes of the DEmiRNAs were predicted, and 118 DEmiRNA-DEG pairs were identified (Figure 6 and Table S5). The *stu-miR156a* and *stu-miR156f* expressions were significantly downregulated under high temperatures in leaves and stems (Figure S8). Among the DEmiRNA–DEG pairs, five *StSPLs* targeted by *stmiR156s* were identified, four of which showed increased expressions under high temperatures (Figure 6 and Figure S9). This result suggests a conservative function of *miR156s*/*SPLs* in plants under high-temperature stress. *miR172* functions as a positive regulator in tuberization through upregulating the *StBEL5* expression [18,19]. However, only two endoglucanase genes targeted by *stumiR172b_5p* were identified (Figure 6), suggesting different regulatory mechanisms of *stu-miR172* under high temperatures. For stems, 150 DEmiRNA–DEG pairs were identified, most of which differed from those in leaves. For instance, only two *StSPLs* targeted by *stu-miR156f-5p* and *stu-miR156a_L* overlapped with those in the leaf (Figure 6 and Figure 7). Interestingly, *miR156* overexpression reduces tuber yield [19,39]. However, a reduction in the *miR156* expression also displayed the suppression of tuberization under high temperatures. Further research is needed on the regulatory mechanism of *miR156* in tuber formation.

Gibberellin is a negative regulator during tuberization by constraining cortical microtubule reorganization at the sub-apical region of the stolon [35,40,41]. Our study identified that a gibberellin deactivator *StCPY714* targeted by *stu-miR8030-5p* was downregulated under high temperatures. This result provides a new clue for the gibberellin involved in tuberization. Moreover, two MADS-box transcription factors, *StAGL8a* and *StAGL8b*, targeted by *stu-miR7981f-p5* and *stu-miR10532A*, showed reduced expression levels under high temperatures (Figure 6 and Figure S9). The orthologous genes in *Arabidopsis* and tomato regulate floral organ development and fruit ripening [32,33,34]. Therefore, *StAGL8a* and *StAGL8b* might be related to the response to high temperature and tuberization.

## 5. Conclusions

In this study, we analyzed regulatory pathways and constructed a comprehensive miRNA–mRNA regulatory network for tuberization under high temperatures. We identified the conservative function of *miR156*/*StSPL* modules in response to high temperatures and suppressing tuberization. We found three candidate miRNA–mRNA pairs in regulating tuberization under high temperatures, including *stu-miR8030-5p*/*StCPY714*, *stu-miR7981f-p5*/*StAGL8a*, and *stu-miR10532A*/*StAGL8b*. Our study provides new insights into potato tuberization under high temperatures and will facilitate the identification of regulatory genes involved in the response to high temperatures.

## Data Availability

Data are contained within the article and supplementary materials.

## Supplementary Materials

The following supporting information can be downloaded at https://www.mdpi.com/article/10.3390/plants13070998/s1: Supplementary File S1: Figure S1. Correlation analysis between RNA sequencing and qPCR results. Figure S2. Hierarchical cluster analysis of overlapped DEGs between UTL_27 vs. TL_20 and UTL_20 vs. TL_20. The color key on the right shows normalized FPKM values for relative gene expressions. Figure S3. Hierarchical cluster analysis and GO enrichment analysis of overlapped DEGs between UTS_27 vs. TS_20 and UTS_20 vs. TS_20. (**a**) Hierarchical cluster analysis of overlapped DEGs. The color key on the right shows normalized FPKM values for relative gene expressions. (**b**) GO enrichment analysis of overlapped DEGs. The colors of the box represent −log_10_ (*p*-value). The size of the boxes represents gene count for GO terms. Biological process, molecular function, and cellular component terms are presented in blue, red, and yellow, respectively. Figure S4. KEGG enrichment analysis of overlapped DEGs in comparison of UTL_27 vs. TL_20 (**a**) and UTS_27 vs. TS_20 (**b**). The box represents −log_10_ (*p*-value). The size of the boxes represents gene count for KEGG terms. Figure S5. Validation of miRNA sequencing data using qPCR. Figure S6. Quantification of miRNA families. The histogram represents the top 15 families. Figure S7. Hierarchical cluster analysis of DEmiRNA target genes. Hierarchical cluster analysis of DEmiRNA target genes in leaf (a) and stem (c). The color key on the right shows normalized FPKM values for relative gene expressions. GO enrichment analysis of DEmiRNA target genes in leaf (b) and stem (d). The colors of the box represent −log_10_ (*p*-value). The size of the boxes represents gene count for GO terms. Biological process, molecular function, and cellular component terms are presented in blue, red, and yellow, respectively. Figure S8. Expression patterns of *miR156* and *mi172* family members. The color key below shows normalized norm values for relative expressions. Figure S9. Expression patterns of candidate genes related to tuberization at high temperatures. The color key below shows normalized FPKM values for relative gene expressions. Supplementary File S2: Table S1. Summary of RNA sequencing. Table S2. Primer lists of RT-PCR. Table S3. Lists of miRNA families. Table S4. Conservation of the identified miRNA with other species. Table S5. Descriptions of miRNA–DEG pairs related to Figure 6. Table S6. Descriptions of miRNA-DEG pairs related to Figure 7.
