# Supplementary material for: A Comprehensive Interaction Network Constructed Using miRNAs and mRNAs Provides New Insights into Potato Tuberization under High Temperatures
Source: Plants (Basel). 2024 Mar 30;13(7):998. doi: 10.3390/plants13070998 (PMC11013713; doi:10.3390/plants13070998)
Supplement: Supplementary file 1 [file plants-13-00998-s001.zip › sup figures proof-reading.pdf]

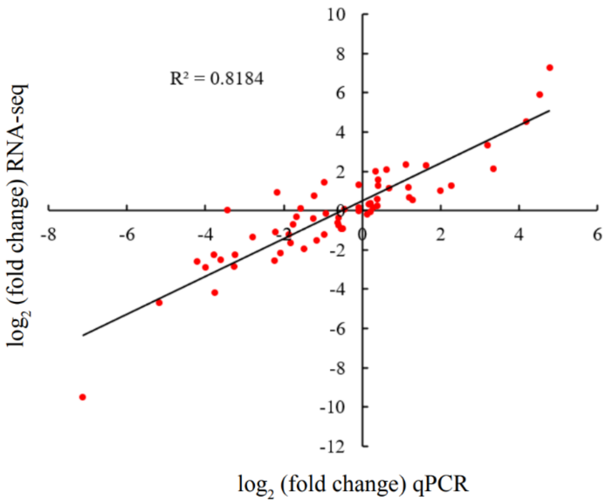

Figure S1. Correlation analysis between RNA sequencing and qPCR results.

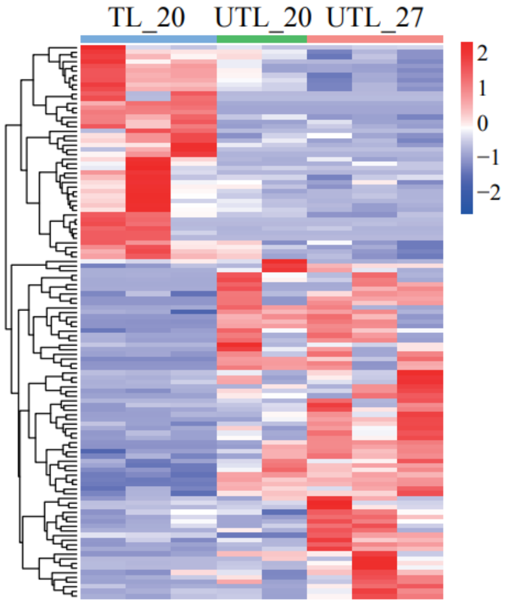

Figure S2. Hierarchical cluster analysis of overlapped DEGs between UTL_27 vs TL_20 and UTL_20 vs TL_20. The color key at the right shows normalized FPKM values for relative gene expression.

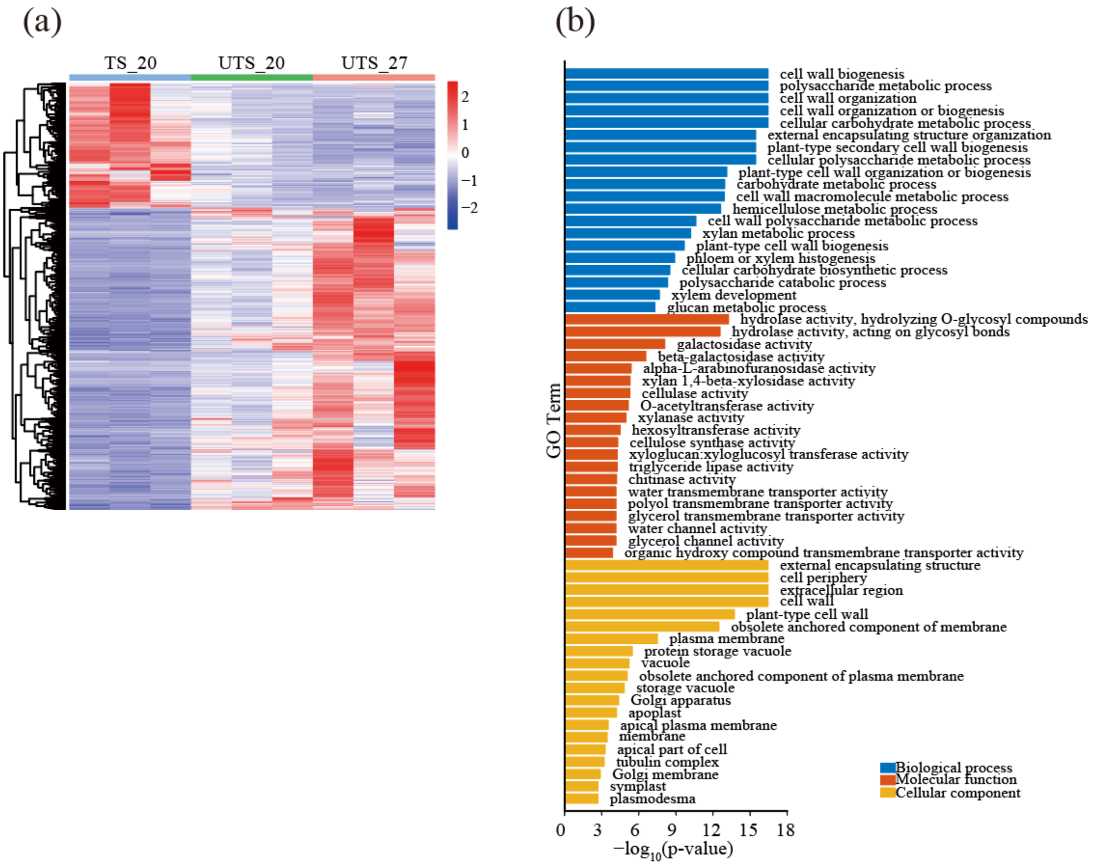

Figure S3. Hierarchical cluster analysis and GO enrichment analysis of overlapped DEGs between UTS_27 vs TS_20 and UTS_20 vs TS_20. **(a)** Hierarchical cluster analysis of overlapped DEGs. The color key at the right shows normalized FPKM values for relative gene expression. **(b)** GO enrichment analysis of overlapped DEGs. The colors of the box represent $-\log_{10}$ (p-value). The size of the boxes represents gene count for GO terms. Biological process, molecular function, and cellular component terms were represented in blue, red, and yellow, respectively.

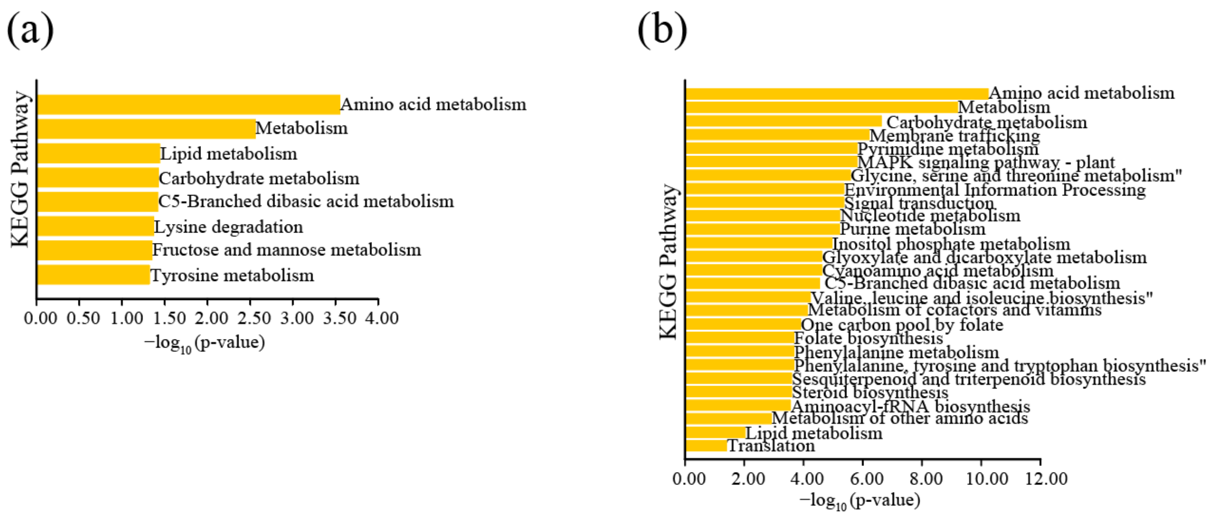

Figure S4. KEGG enrichment analysis of overlapped DEGs compared of UTL_27 vs TL_20 **(a)** and UTS_27 vs TS_20 **(b)**. The box represents $-\log_{10}$ (p-value). The size of the boxes represents gene count for KEGG terms.

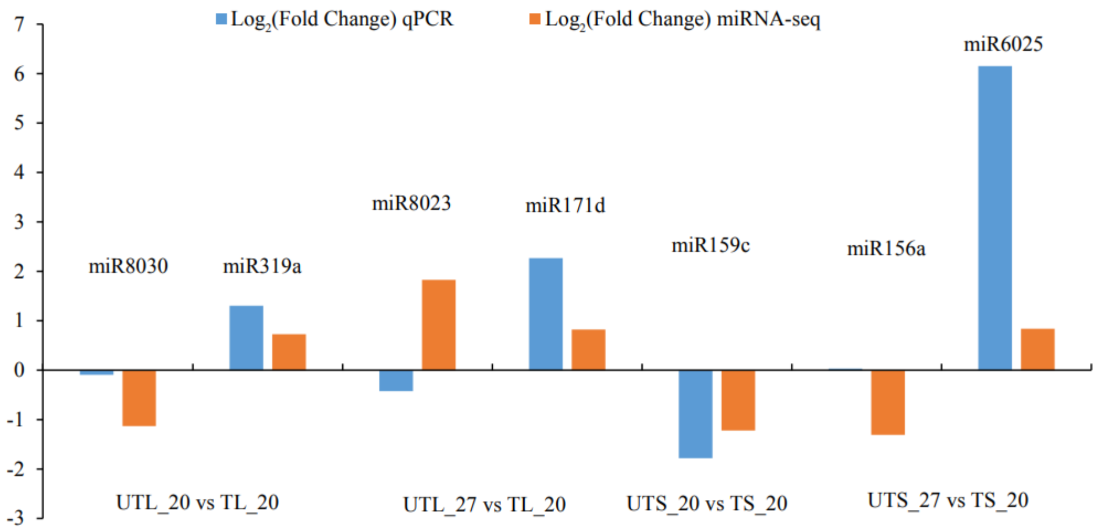

Figure S5. Validation of miRNA sequencing data using qPCR.

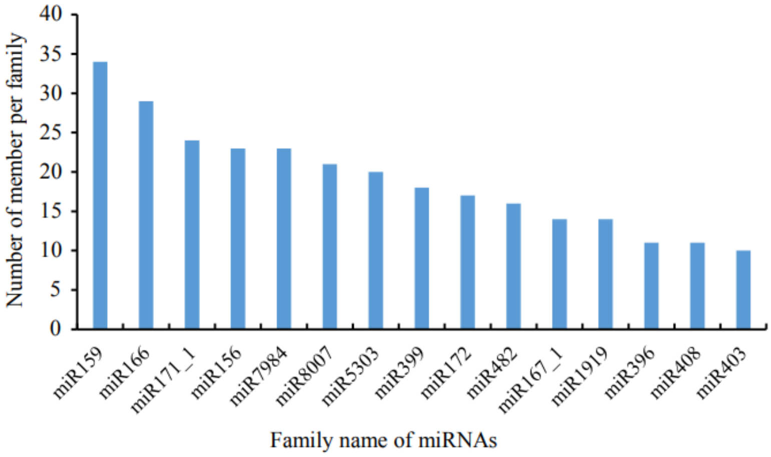

Figure S6. Quantification of miRNA families. The histogram represents the top 15 families.

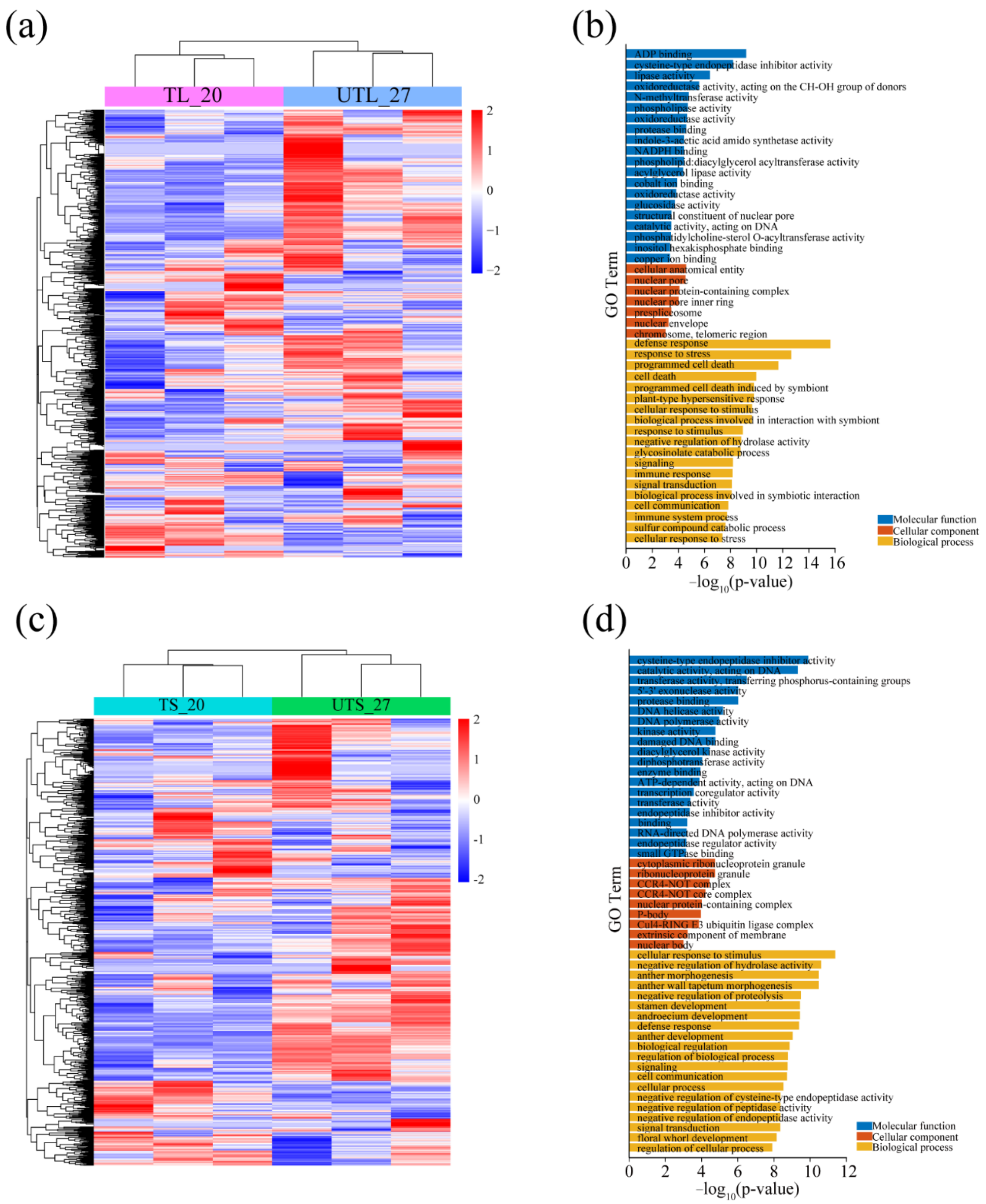

Figure S7. Hierarchical cluster analysis of DEmiRNA target genes. Hierarchical cluster analysis of DEmiRNA target genes in leaf (a) and stem (c). The color key at the right shows normalized FPKM values for relative gene expression. GO enrichment analysis of DEmiRNA target genes in leaf (b) and stem (d). The colors of the box represent −log$_{10}$ (p-value). The size of the boxes represents gene count for GO terms. Biological process, molecular function, and cellular component terms were represented in blue, red, and yellow, respectively.

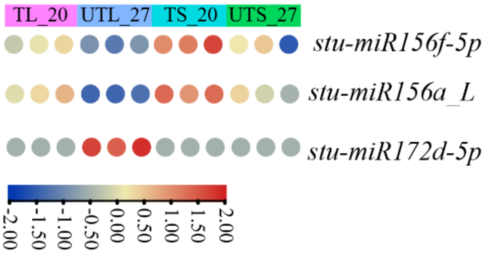

Figure S8. Expression patterns of *miR156* and *mi172* family members. The color key below shows normalized norm values for relative expression.

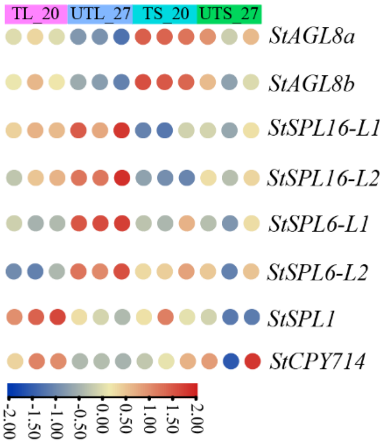

Figure S9. Expression patterns of candidate genes related to tuberization at high temperatures. The color key below shows normalized FPKM values for relative gene expression.
